# Supplementary material for: Shades of grey: host phenotype dependent effect of urbanization on the bacterial microbiome of a wild mammal
Source: Anim Microbiome. 2021 Jul 5;3:46. doi: 10.1186/s42523-021-00105-4 (PMC8256534; doi:10.1186/s42523-021-00105-4)
Supplement: Supplementary file 1 — Additional file 1: Supplementary tables and figures referenced in the main-text of Shades of grey: host phenotype dependent effect of urbanization on the bacterial microbiome of a wild mammal. [file 42523_2021_105_MOESM1_ESM.docx]

**Supplemental Information for:**

**Shades of grey: Host phenotype dependent effect of urbanization on the bacterial microbiome of a wild mammal**

Mason R. Stothart^1,a^, Amy E.M. Newman^2,b^

^1^Department of Ecosystem and Public Health, Faculty of Veterinary Medicine, University of Calgary, Calgary T2N 4Z6, Canada

^2^Department of Integrative Biology, College of Biological Sciences, University of Guelph, Guelph N1G 2W1, Canada

**Table of Contents:**

| **Table S1** | Page 1 |
| --- | --- |
| **Figure S1** | Page 2 |
| **Figure S2** | Page 3 |
| **Figure S3** | Page 4 |
| **Figure S4** | Page 5 |
| **Figure S5** | Page 6 |
| **Figure S6** | Page 7 |
| **Figure S7** | Page 8 |
| **Figure S8** | Page 9 |

|  | Built-Environment | | | |  | Forest | | | |
| --- | --- | --- | --- | --- | --- | --- | --- | --- | --- |
| Genus | Full Dataset | Agouti | Intermediate | Black |  | Full Dataset | Agouti | Intermediate | Black |
| Alistipes | 1.25% ± 1.34* | 1.17% ± 1.24 | 1.28% ± 1.54* | 1.28% ± 0.91 |  | 2.66% ± 2.23* | 2.94% ± 2.78 | 2.91% ± 1.76* | 1.61% ± 1.39 |
| Bacteroides | 5.93% ± 7.63 | 6.72% ± 5.52* | 6.28% ± 9.37 | 4.15% ± 4.73 |  | 3.33% ± 3.72 | 3.42% ± 3.95* | 3.8% ± 4.08 | 2.22% ± 1.77 |
| Blautia | 0.26% ± 0.27* | 0.34% ± 0.36* | 0.25% ± 0.24 | 0.21% ± 0.2 |  | 0.13% ± 0.15* | 0.15% ± 0.15* | 0.15% ± 0.17 | 0.08% ± 0.06 |
| Clostridiales (sub-group vadinBB60) | 0.33% ± 0.68* | 0.4% ± 0.94 | 0.23% ± 0.27* | 0.45% ± 0.89 |  | 0.76% ± 1.15* | 0.47% ± 0.66 | 0.97% ± 1.33* | 0.89% ± 1.37 |
| Coprococcus (sub-group 3) | 0.08% ± 0.08* | 0.06% ± 0.04 | 0.08% ± 0.09 | 0.09% ± 0.08 |  | 0.04% ± 0.05* | 0.06% ± 0.06 | 0.04% ± 0.03 | 0.02% ± 0.03 |
| Eisenbergiella | 0.05% ± 0.05* | 0.05% ± 0.05* | 0.06% ± 0.06 | 0.03% ± 0.03 |  | 0.03% ± 0.05* | 0.02% ± 0.02* | 0.04% ± 0.07 | 0.02% ± 0.04 |
| Lachnoclostridium | 0.28% ± 0.39* | 0.36% ± 0.47* | 0.25% ± 0.4 | 0.22% ± 0.23 |  | 0.11% ± 0.1* | 0.13% ± 0.13* | 0.1% ± 0.07 | 0.08% ± 0.07 |
| Lachnospiraceae (sub-group NK4A136) | 2.31% ± 2.99* | 1.86% ± 3.14 | 2.01% ± 3.03 | 3.54% ± 2.29 |  | 3.71% ± 3.6* | 4.04% ± 4.24 | 3.26% ± 2.91 | 3.96% ± 3.39 |
| Lachnospiraceae (sub-group FCS020) | 0.07% ± 0.09* | 0.04% ± 0.05 | 0.07% ± 0.09 | 0.08% ± 0.11 |  | 0.13% ± 0.15* | 0.13% ± 0.13 | 0.14% ± 0.19 | 0.1% ± 0.07 |
| Lachnospiraceae (sub-group 006) | 0% ± 0.01 | 0.01% ± 0.01* | 0% ± 0.01 | 0.01% ± 0.02 |  | 0% ± 0 | 0% ± 0* | 0% ± 0 | 0% ± 0 |
| Prevotella (sub-group 9) | 1.78% ± 5.75* | 1.93% ± 7.24 | 2.33% ± 5.95* | 0.35% ± 0.9 |  | 0.08% ± 0.36* | 0.15% ± 0.52 | 0% ± 0.01* | 0.08% ± 0.3 |
| Parasutterella | 0.52% ± 0.55* | 0.57% ± 0.76* | 0.54% ± 0.48 | 0.41% ± 0.31 |  | 0.26% ± 0.24* | 0.27% ± 0.25* | 0.25% ± 0.22 | 0.28% ± 0.28 |
| Oxalobacter | 0.17% ± 0.28* | 0.16% ± 0.13* | 0.18% ± 0.26 | 0.14% ± 0.12 |  | 0.08% ± 0.07* | 0.06% ± 0.05* | 0.09% ± 0.09 | 0.1% ± 0.06 |
| Mollicutes (sub-group RF39) | 1.59% ± 1.22* | 1.75% ± 1.54* | 1.55% ± 1.08 | 1.46% ± 1.03 |  | 1.08% ± 1.15* | 1% ± 0.79* | 1.15% ± 1.3 | 1.1% ± 1.39 |
| Lactobacillus | 8.32% ± 12.71 | 12.48% ± 14.62* | 7.26% ± 12.68 | 5.52% ± 8.11 |  | 3.81% ± 8.56 | 1.8% ± 3.77* | 3.31% ± 6.31 | 8.67% ± 14.9 |
| Ruminiclostridium (sub-group 5) | 0.11% ± 0.16* | 0.11% ± 0.15 | 0.12% ± 0.19 | 0.08% ± 0.09 |  | 0.04% ± 0.08* | 0.05% ± 0.09 | 0.03% ± 0.08 | 0.03% ± 0.04 |
| Ruminococcaceae (sub-group 014) | 3.48% ± 2.13* | 3.53% ± 1.7* | 3.69% ± 2.38 | 2.92% ± 1.93 |  | 2.37% ± 1.91* | 2.06% ± 1.24* | 2.85% ± 2.32 | 2.02% ± 1.84 |
| Eggerthellaceae (unclassified genera) | 0.04% ± 0.05* | 0.04% ± 0.06* | 0.04% ± 0.06 | 0.03% ± 0.03 |  | 0.02% ± 0.02* | 0.02% ± 0.02* | 0.02% ± 0.02 | 0.01% ± 0.01 |
| Clostridiales (sub-group XIII) | 0.01% ± 0.02 | 0.01% ± 0.02* | 0.01% ± 0.01 | 0.01% ± 0.01 |  | 0% ± 0.01 | 0% ± 0.01* | 0.01% ± 0.01 | 0% ± 0.01 |
| Ruminococcaceae (sub-group 008) | 0.1% ± 0.2* | 0.08% ± 0.16 | 0.1% ± 0.17* | 0.11% ± 0.29 |  | 0.05% ± 0.17* | 0.08% ± 0.26 | 0.02% ± 0.03* | 0.03% ± 0.03 |
| Ruminococcaceae (unclassified genera) | 5.72% ± 3.83 | 5.43% ± 3.7* | 5.35% ± 3.82 | 6.89% ± 3.76 |  | 4.11% ± 2.22 | 4.08% ± 1.99* | 4.36% ± 2.49 | 3.68% ± 1.97 |
| Lachnospiraceae (unclassified genera) | 25.38% ± 11.4* | 22.05% ± 8.92* | 25.02% ± 12.88 | 30.35% ± 8.54 | | 30.86% ± 11.57* | 33.6% ± 12.39* | 27.54% ± 9.54 | 32.22% ± 11.98 |

Table S1: Average percent abundance (± SD) of bacterial genera between environments, separated by phenotype.

* denotes genera which among colour phenotypes, significantly differed between environments (based upon ANCOM-BC tests).


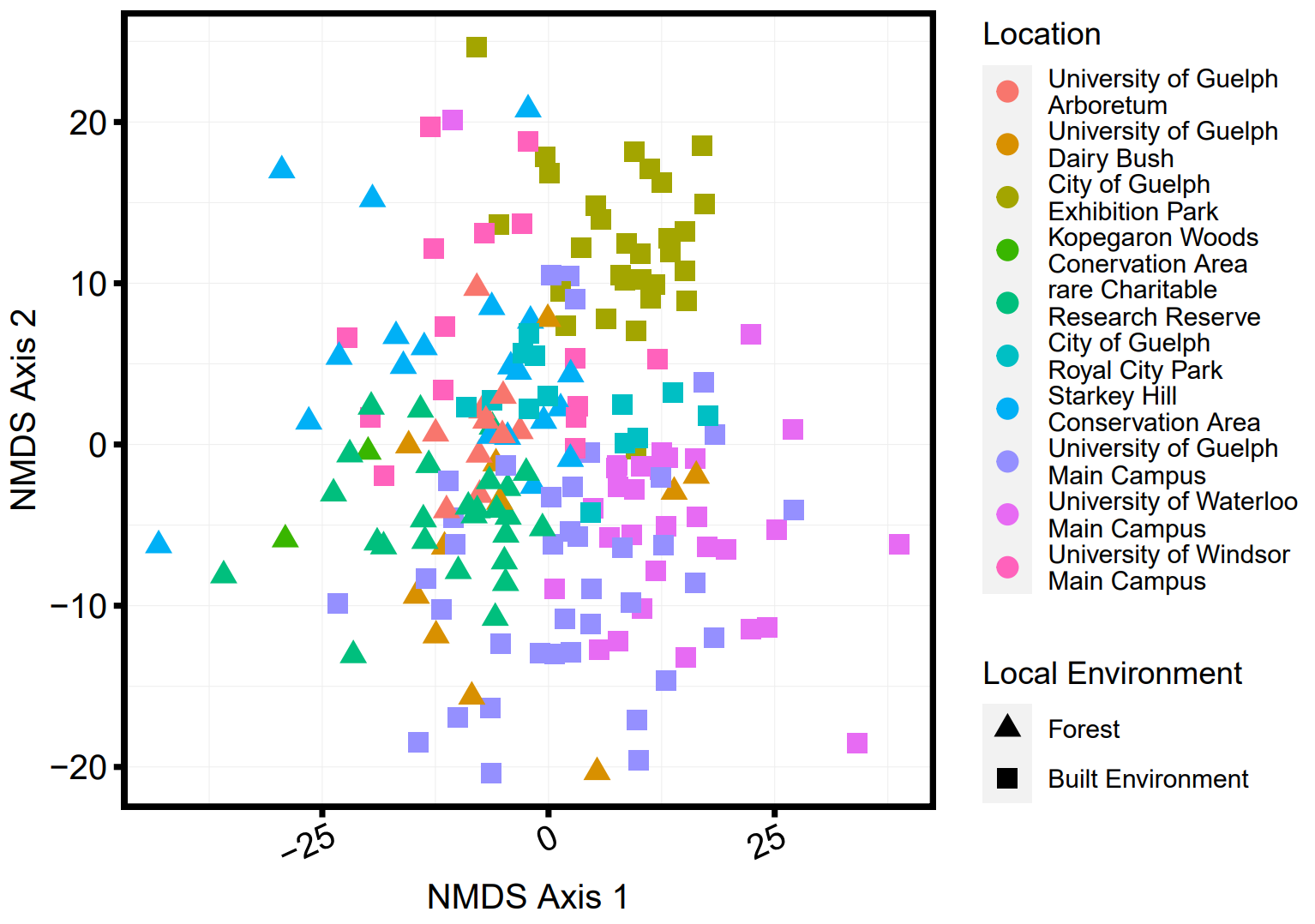


Figure S1: A non-metric multi-dimensional scaling ordination of the eastern grey squirrel fecal bacterial microbiome, coloured by sampling site and shaped by local environment type (stress value = 0.23).


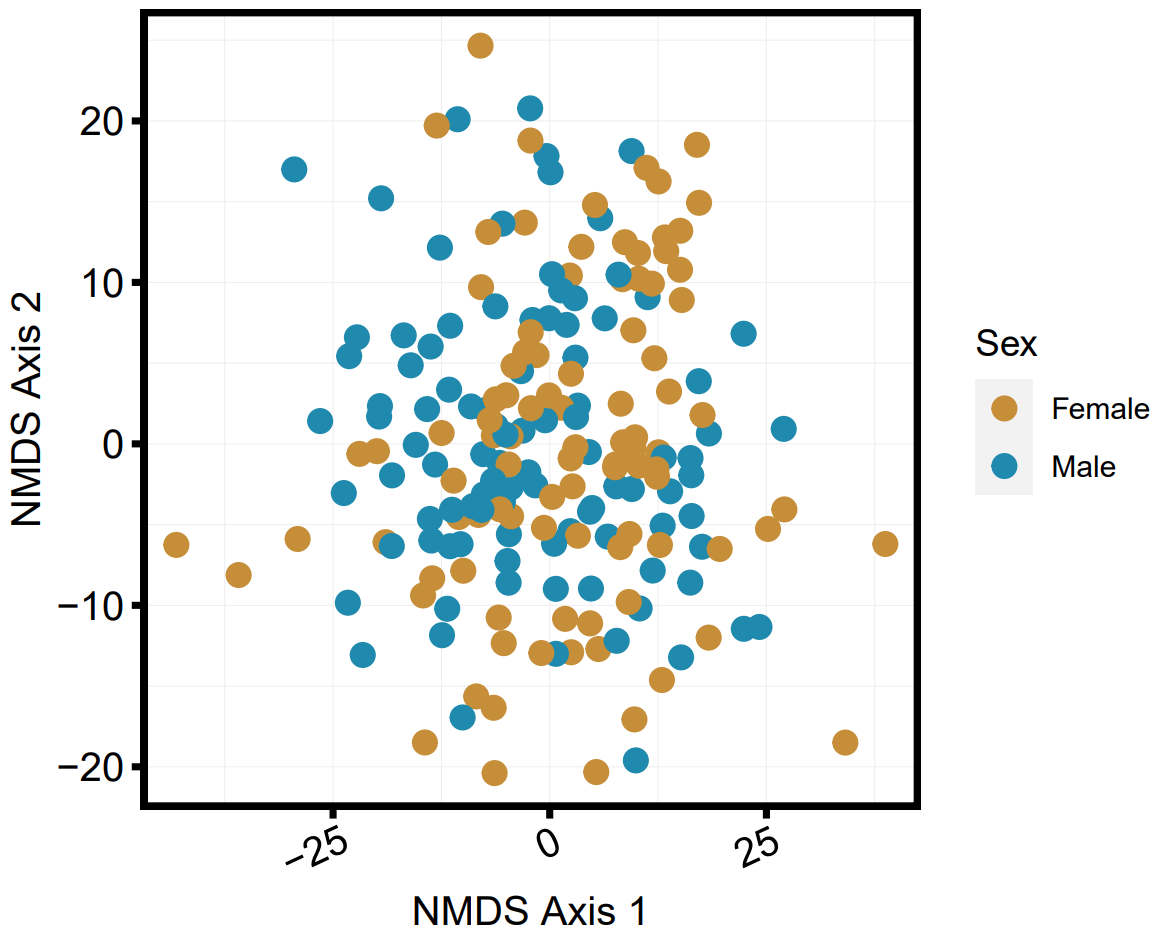


Figure S2: A non-metric multi-dimensional scaling ordination of the eastern grey squirrel fecal bacterial microbiome, coloured by sex (stress value = 0.23).


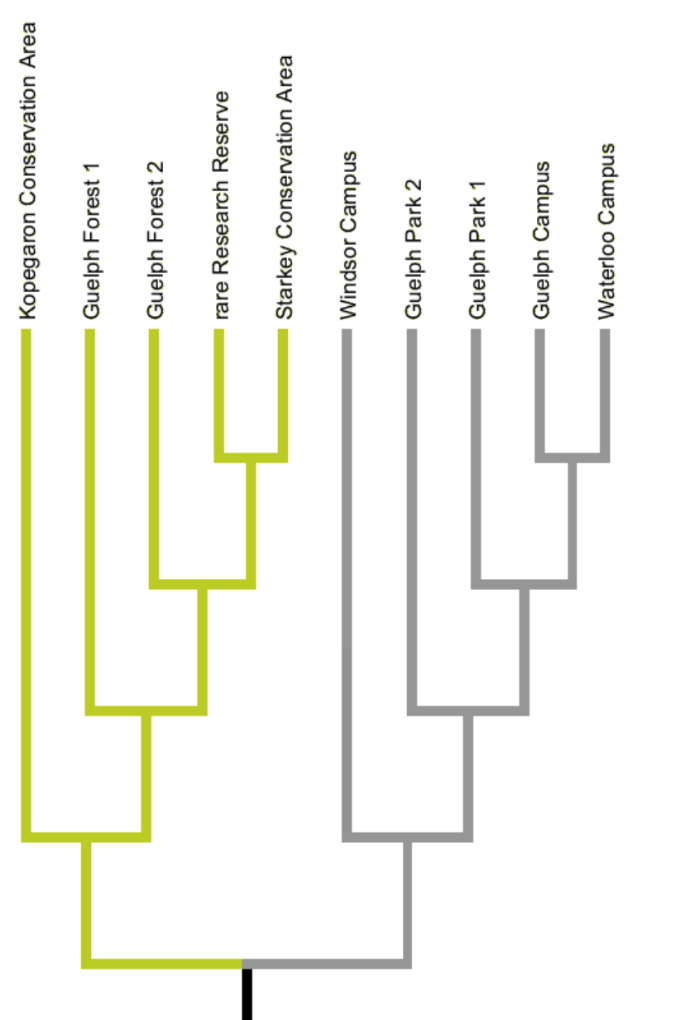


Figure S3: A hierarchical clustering diagram of site pooled samples of the eastern grey squirrel bacterial microbiome, created from centred log-ratio transformed OTU counts and using a UPGMA method. Green branches denote forest sites while grey branches denote built-environment sites.


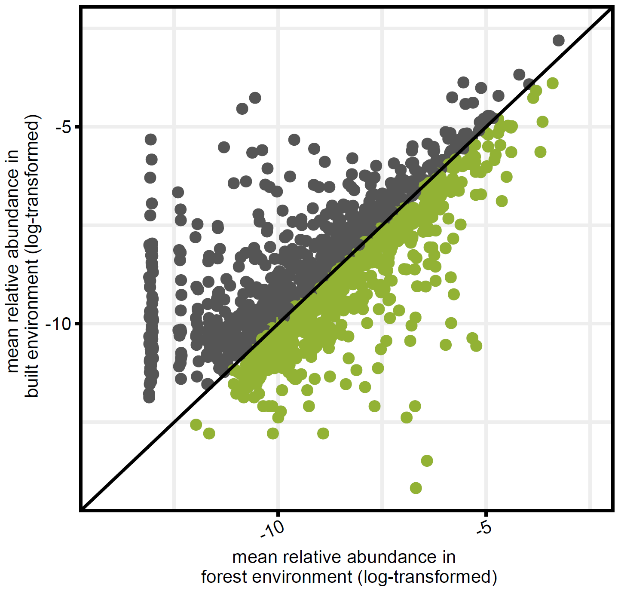


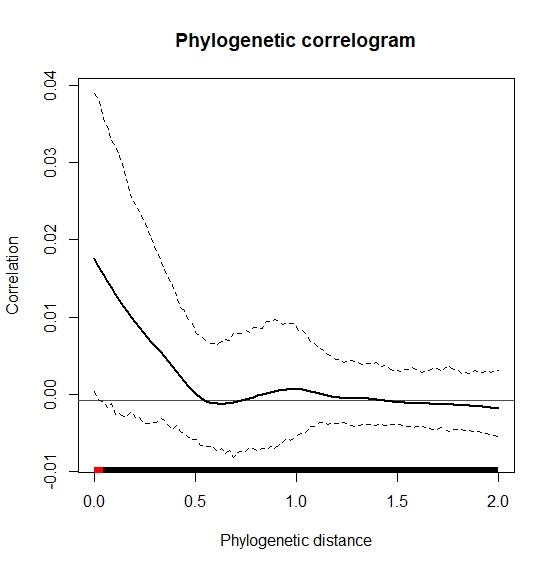


Figure S4: Plots of (A) log-transformed mean relative abundance of OTUs (points) among forest (●) versus built-environment (●) squirrels with 1:1 line (solid black) and (B) a phylocorrelogram of OTU deviation from the 1:1 line, which shows a positive phylogenetic signal over short phylogenetic distances (significance identified by a red line) for bacterial OTU bias towards squirrel local environment type.


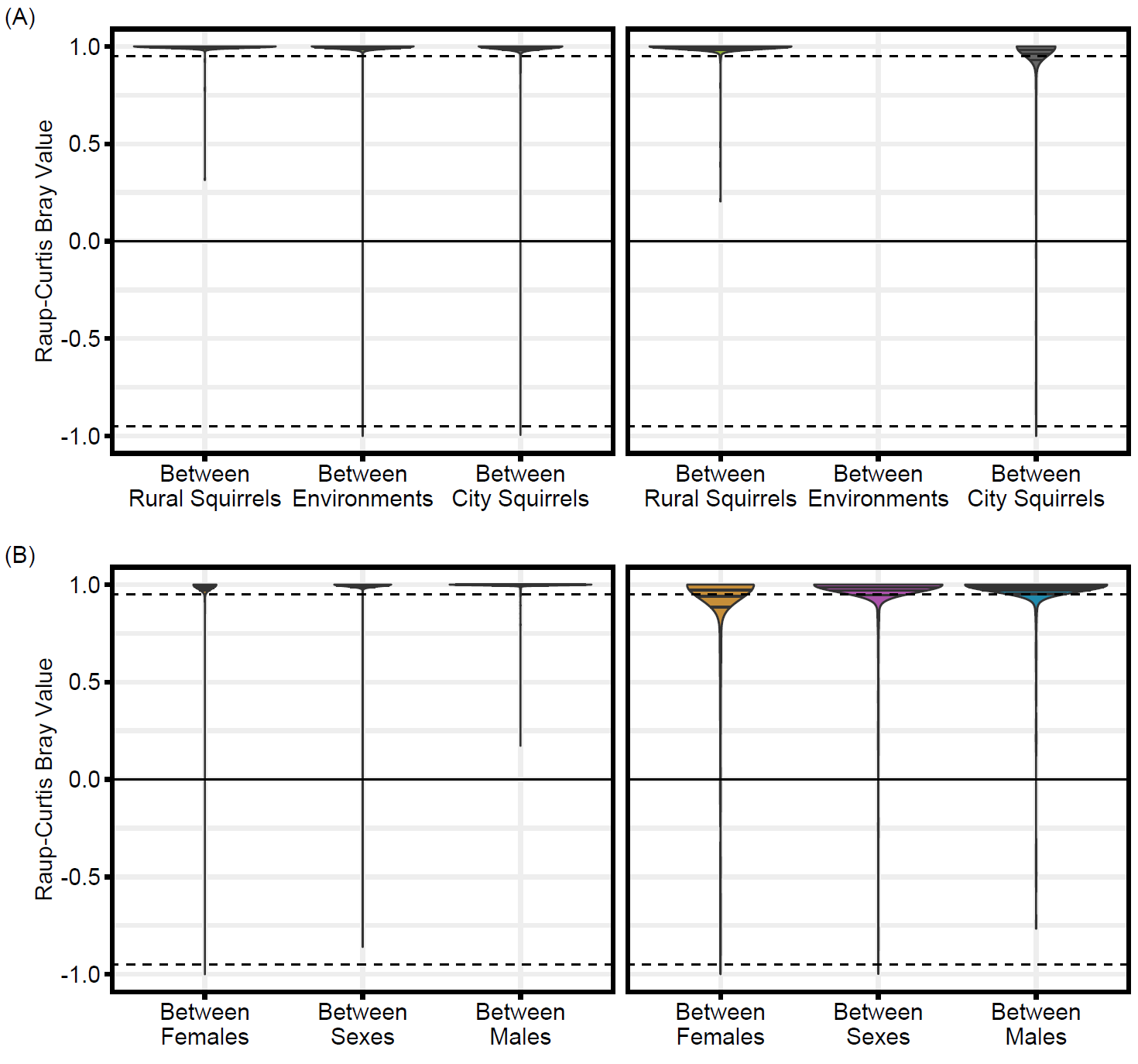


Figure S5: Plots of Raup-Crick_bray_ values within and between (A) environments and (B) sexes, facetted by pairwise comparisons made between sites (left) versus within sites (right).

Figure S6: Boxplot, AUC-ROC curve, and density plots from selection balance analyses used to discriminate between forest and urban microbiomes at the bacterial taxonomic level of a) genus and b) family.


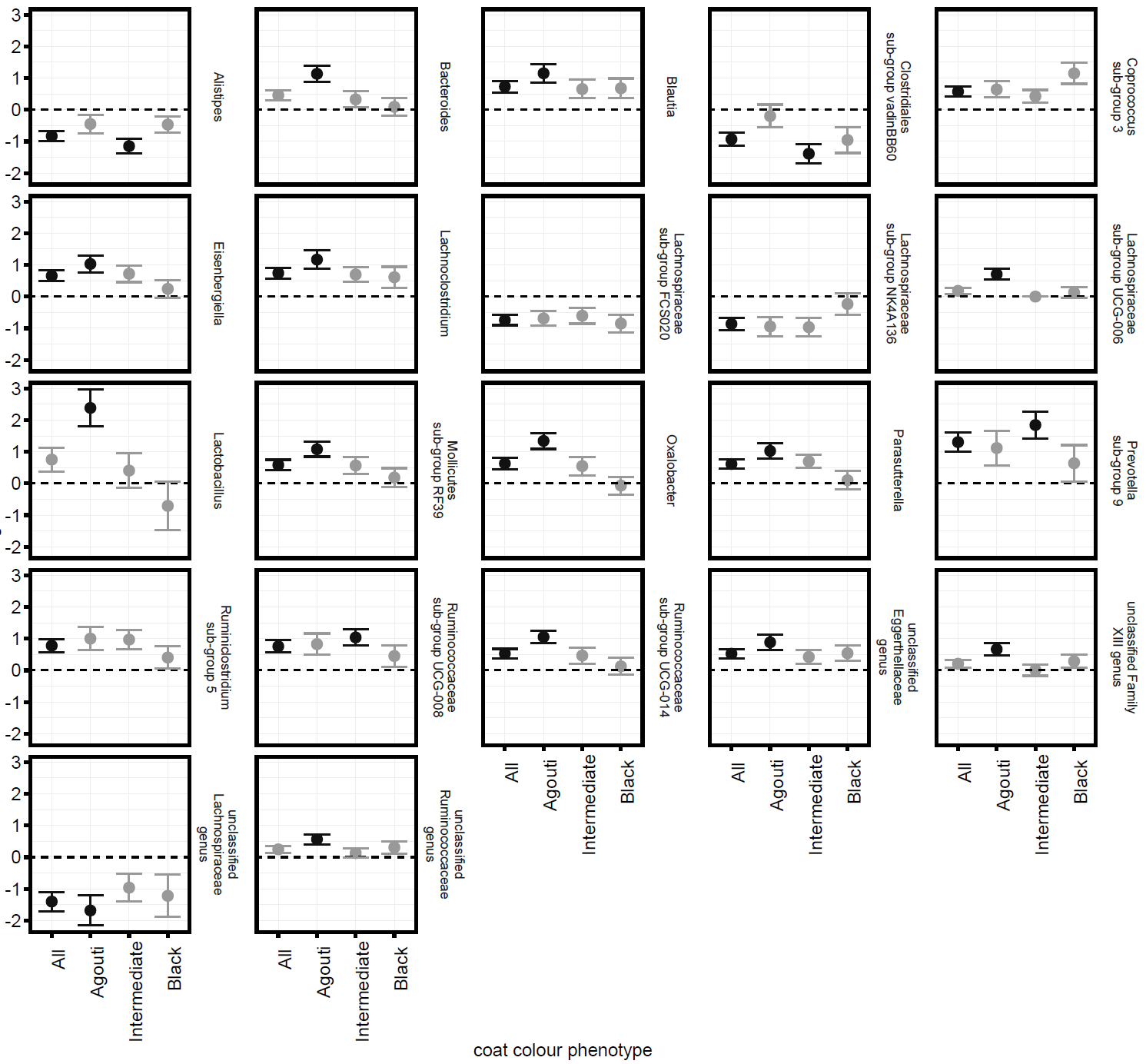


Figure S7: Forest plots of ANCOM-BC estimated log difference (dot) and standard error (whiskers) of genus abundances which significantly differed (black) or did not differ (grey) between forest (y-values < 0) and built-environment (y-values > 0) squirrels of pooled, or parsed coat colour phenotypes.


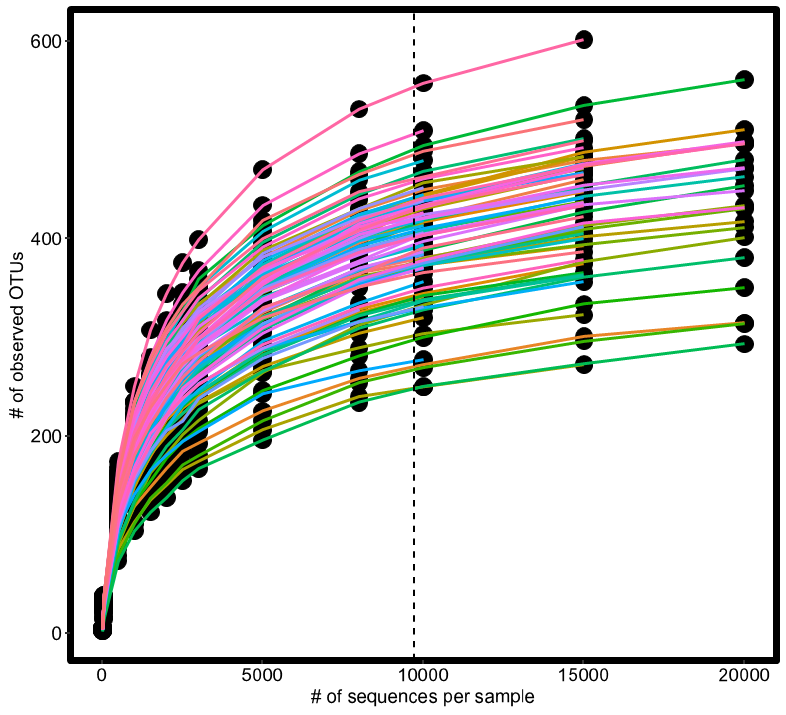


Figure S8: A rarefaction curve of observed # of OTUs versus sub-sampled sequencing depth. Coloured lines represent separate individuals. The dotted line demarcates level of rarefaction used for analyses.
